# The INTEGRITY guidelines – ethical guidance for research involving deceased donation and transplantation

**DOI:** 10.3389/ti.2026.17151

**Published:** 2026-09-18

**Authors:** Dominique E. Martin, Ali Abbasi, Riadh A. S. Fadhil, John L. R. Forsythe, Matthieu Le Dorze, Jayme E. Locke, Elmi Muller, Gabriel C. Oniscu, Helen Opdam, Karen Rockell, Marion J. Siebelink, Shih-Ning Then, Emma Tumilty, INTEGRITY Project Collaborator Team, Juntaro Ashikari, Pacifico Calderon, Elena Cavazzoni, Celeste De Vaal, Beatriz Domínguez-Gil, Rosalie Grivell, Daniel Harvey, Marisa Herson, Alison Hodak, Georgina Irish, Victoria Jennings, Wenshi Jiang, Marta López-Fraga, Heather Machin, Francisco Martinez, María Victoria Martínez-López, Olivia Ngan, Jeffrey P. Orlowski, Brendan Parent, Rebecca Pentz, Alicia Perez Blanco, Rizmina Rilwan, Sibele Schuantes, David Thomson, Sandra Venter, Matthew J. Weiss, Matthew Wellberry-Smith

**Affiliations:** 1 School of Medicine, Deakin University, Geelong, VIC, Australia; 2 Department of Surgery, University of California San Francisco, San Francisco, CA, United States; 3 Hamad Medical Corporation, Doha, Qatar; 4 Weill Cornell Medicine - Qatar, Doha, Qatar; 5 Department of Surgery, The University of Edinburgh, Edinburgh, United Kingdom; 6 Department of Anesthesia and Critical Care Medicine, Hopital Lariboisiere, Paris, France; 7 Department of Surgery, New York University Grossman School of Medicine, New York, NY, United States; 8 Department of Surgery, Faculty of Medicine and Health Sciences, Stellenbosch University, Stellenbosch, South Africa; 9 Division of Transplantation Surgery, Department of Clinical Science, Intervention and Technology, Karolinska Institutet, Stockholm, Sweden; 10 Department of Intensive Care, Austin Hospital, Heidelberg, VIC, Australia; 11 Australian Organ and Tissue Authority, Canberra, ACT, Australia; 12 UK Organ Donation and Transplantation Research Network C.I.C., Peterborough, United Kingdom; 13 UMCG Transplantatiecentrum, Universitair Medisch Centrum Groningen, Groningen, Netherlands; 14 Australian Centre for Health Law Research, Queensland University of Technology, Brisbane, QLD, Australia; 15 First Nations Health Unit, The Royal Melbourne Hospital, Melbourne, VIC, Australia

**Keywords:** deceased donation, ethics of clinical research, guidelines, research ethics, transplantation

## Abstract

The absence of ethical guidance for research that involves actual and prospective deceased donors of organs, cells or tissues for transplantation presents challenges for researchers, donation agencies, and participants in such research. To address this, the European Society for Organ Transplantation (ESOT), together with the International Society for Organ Donation Professionals (ISODP) and The Transplantation Society (TTS), convened an interdisciplinary, multinational team to develop the first international guidelines for ethical practice in research involving deceased donation and transplantation activities (INTEGRITY). The guidelines were developed over twelve months via an international, iterative consultative process. The resultant principles are presented in this article.

## Introduction

The donation of human organs, cells, and tissues after death for use in therapeutic transplantation often presents ethical complexities due to the intersection of end-of-life care with opportunities for donation. Foremost among these are concerns for the validity of decision-making about donation and respect for the wishes of the prospective donor, for the wellbeing of donors prior to death, and for the wellbeing of their families throughout the end-of-life period [1]. The allocation of donated organs and tissues for use in transplantation further entails concern for equity of access to these scarce resources [2], and ethical challenges can also arise in decision-making by organ transplant candidates about acceptance of offered organs from deceased donors [3].

These ethical challenges are increasingly complicated by the addition of research considerations, as efforts to optimise the recovery and utilisation of organs and tissues in transplantation require trials of new interventions involving deceased donors or donated organs and tissues [4]. There is also growing recognition that individuals in whom donation for transplantation after death is considered represent a valuable potential source of organs, cells and tissues that may be used in various research activities beyond the field of transplantation [5]. Relevant research activities range from pre- or post-mortem donor or donor-organ intervention studies aimed at improving utilisation of organs for transplantation, to use of donor tissue samples in basic sciences research, investigation of family experiences of and decision-making about deceased donation, studies evaluating the properties or outcomes of tissue grafts, and many more [6–13].

Ethical guidance for deceased donation for the purpose of transplantation is well-established at the international level [14], as is ethical guidance for research that involves living persons [15]. However, there is little guidance available to support the ethical design and conduct of research that involves people for whom donation for transplantation is considered during the end-of-life period (henceforth ‘donors’) or recipients of transplants obtained from deceased donors (henceforth ‘recipients’). In the context of deceased donation, the end-of-life period typically extends from the time when death is first anticipated, throughout the dying process and beyond the determination of death. This period may encompass both pre- and also post-mortem interventions in the body of the donor. Following the end-of-life period, materials donated by a deceased donor or data relating to a donor may be used in research even several years later, for example when studying the long-term outcomes of deceased donor transplantation. Ethical guidance for biobanks may address post-mortem use of materials and data but is typically designed to support research use of materials donated by living persons or obtained after death in accordance with an advance directive [8, 9].

Consequently prospective researchers, transplant clinicians, and professionals involved in deceased donation for transplantation (henceforth ‘donation professionals’) face considerable difficulties when developing, evaluating or implementing studies that intersect in various ways with deceased donation for transplantation [16–21]. Some guidance has addressed specific types of research involving deceased donors, such as research involving the recently deceased and donor-intervention studies [20, 22], or regulatory frameworks in specific jurisdictions [23]. However, no comprehensive guidance has been developed that considers the full range of ethical considerations pertinent to the diverse research activities that may indirectly or directly involve donors [16]. Consequently, uncertainty regarding ethical standards and governance processes presents risks of harm for those involved in or impacted by research activities and may also impede or prevent valuable research.

To address this problem, the European Society for Organ Transplantation (ESOT), together with the International Society for Organ Donation Professionals (ISODP) and The Transplantation Society (TTS), convened an interdisciplinary, multinational team to develop the first international guidelines for ethical practice in research involving deceased donation and transplantation activities (INTEGRITY). In this paper, we report on the INTEGRITY Project, outlining the development process, briefly discussing the significance of the eight core ethical principles which underpin the full guidelines, and presenting the complete set of principles. The complete guidelines are accessible here [https://esot.org/the-integrity-project/] in several languages.

## Development of the guidelines

The guidelines were developed over 12 months via an international, iterative consultative process (see Figure 1). This approach was designed to explore potential ethical considerations from a wide range of perspectives and experiences, as these considerations may be significantly impacted by sociocultural, economic and health system factors in the local context in which research takes place. The development process can be understood in four main phases: initial scoping, iterative drafting with expert input, public consultation, and final validation.

**FIGURE 1:**
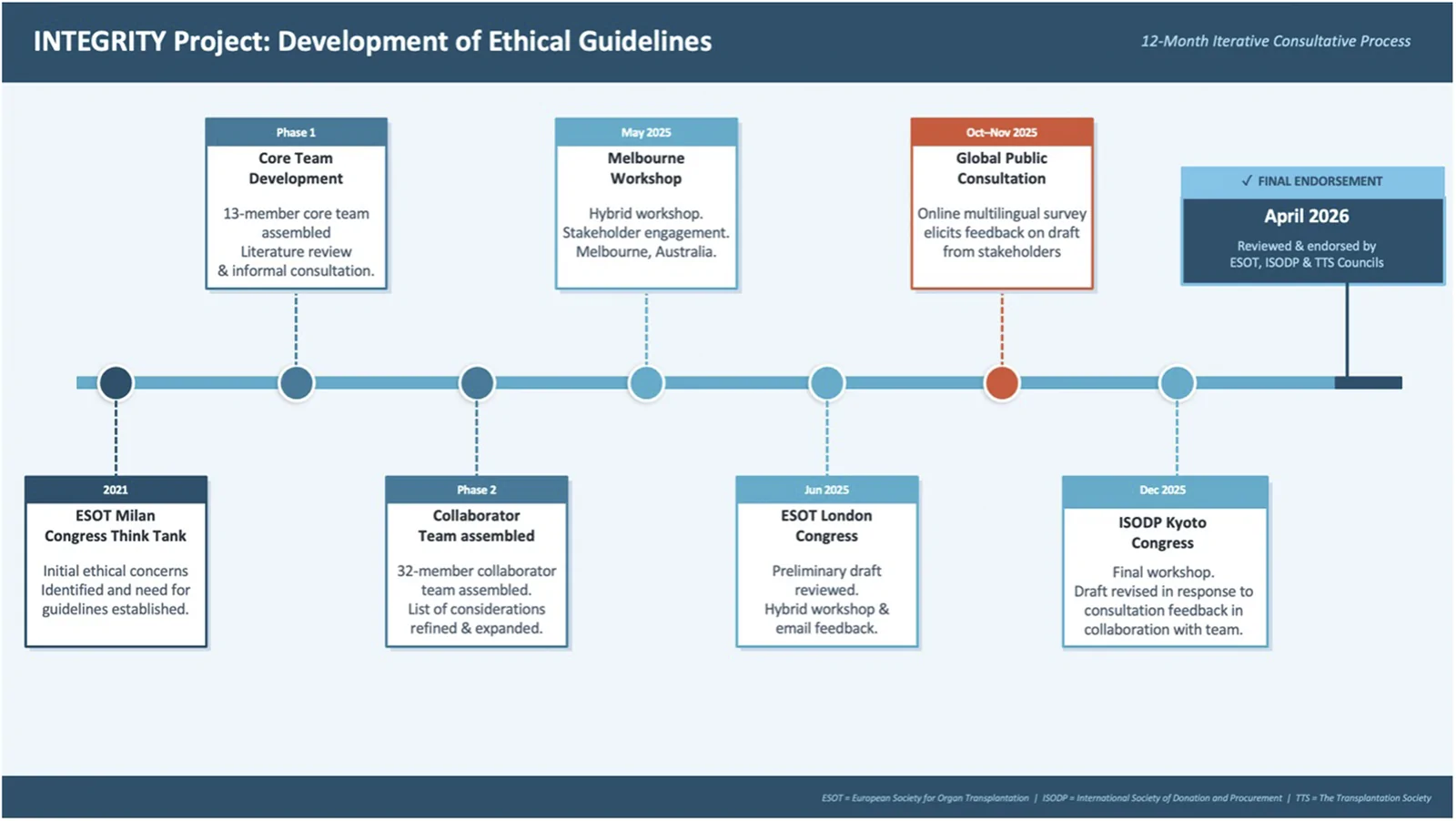
Development process for the INTEGRITY Guidelines.

### Initial scoping

Building on the work of a ‘think tank’ group assembled by ESOT as part of the 2021 Milan Congress [16], an initial set of ethical concerns and questions of specific relevance to various types of research involving deceased donation was developed via review of the literature and targeted consultation with members of the ‘core’ 13 member INTEGRITY Project Team. This international group comprised individuals with diverse experience and expertise in health law and ethics, research and clinical practice involving deceased donation and transplantation, and lived experience of transplantation. The resultant list was further expanded and refined in consultation with the larger 27 member ‘collaborator’ team representing a wider range of expertise from around the world, and with stakeholders who participated in a hybrid workshop held in Melbourne, Australia in May 2025.

### Iterative drafting

An initial draft of the guidelines was prepared by members of the core team and revised in response to feedback from collaborators received during a hybrid workshop held during the ESOT Congress in London, United Kingdom in June 2025, and via email circulation.

### Public consultation

A further draft was developed, which was made available for public consultation between 2nd October and 7th November 2025, via an anonymous online survey hosted on the Research Electronic Data Capture software platform [24]. Participation was possible in English, Arabic, Chinese, French, Italian, Japanese, Portuguese and Spanish. The text of the guidelines and the survey was initially translated using Claude (https://claude.ai), with all translations subsequently reviewed and validated by native speakers with professional experience in transplantation or ethics. In addition to some basic demographic questions, the feedback questionnaire requested levels of agreement with the proposed principles of the draft guidelines using a five-point Likert scale and respondents had the option of providing comments to clarify their views or propose additions or revisions.

Any person aged 18 years or older was eligible to participate in the consultation. Participation was encouraged via social media posts on LinkedIn and X and email communications to the members of ESOT, ISODP, and TTS, and by email dissemination among collaborator networks. National and regional professional societies for transplantation and donation, as well as research ethics organizations and representatives of stakeholders such as the World Health Organization (WHO) and the World Medical Association were also contacted via email to invite their participation.

### Final validation

The results of the consultation survey were used to guide further revisions to the draft guidelines by the core project team, which were again reviewed and further revised in response to feedback from the collaborator team. A final workshop was held in Kyoto, Japan during the ISODP Congress in December 2025 at which further feedback was received. The final version was reviewed and endorsed by the ESOT, ISODP, and TTS Councils in April 2026.

## Results of the public consultation

271 complete responses were submitted during the official consultation period, by respondents from 48 countries. Respondents represented a broad range of countries and professional backgrounds, reflecting the diversity of perspectives included in the consultation process. A majority were female (53%; male 44%; 3% non-binary or undisclosed) and identified as health professionals (54%; 13% were non-clinical organ, cell or tissue banking professionals; 10% ethicists, legal scholars, or members of ethics committees; 6% researchers; 4% donor family members or transplant recipients; 11% other including members of the public). No proposed draft principle received less than 75% agreement (including agree and strongly agree responses), and 74% of the statements including the proposed core principles, received at least 90% agreement.

## The INTEGRITY guidelines

The Guidelines comprise a preamble that defines their scope, a set of eight core ethical principles under which several subprinciples are outlined that provide further specification, and a glossary that defines key terms used in the guidelines.

### Purpose and scope

The guidelines are intended to assist researchers, transplantation and donation professionals, prospective research participants, donation decision-makers (including surrogates making a decision on behalf of a possible donor), and ethics governance bodies in designing, evaluating and conducting research that involves deceased donors or use of organs, cells, or tissues from deceased donors. The guidelines apply to research involving adults and children, including persons who lack decision-making capacity. They are applicable to a range of research activities including studies that involve:

1. Interventions in the body of the deceased donor.
2. Collection and/or use of personal information from or about the deceased donor.
3. Removal, interventions in, and/or use of organs, cells, or tissues donated after death—including prior to transplantation, use of stored materials, and studies of transplant recipients.
4. Collection and use of information from donor families, recipients of transplants from deceased donors, or healthcare professionals about their lived experiences of deceased donation.
5. Secondary use of information about deceased donors or donated materials originally collected for clinical or quality assurance purposes.

### Use and limitations of the guidelines

All human research activities require ethical governance, including those that intersect with deceased donation for transplantation. As noted in Principle 1.6, “All research involving deceased donors should be subject to scientific and ethical review and oversight at an appropriate level.” The ethical oversight of deceased donation and transplantation provides a reassuring framework for the protection of donors and transplant recipients. However, research activities require specific consideration of ethical elements that are not within the scope of clinical practice in donation and transplantation. On the other hand, many of the ethical complexities of research that intersects with deceased donation may be unfamiliar to individuals or groups responsible for the ethical oversight of human research.

The INTEGRITY Guidelines are intended to help bridge the gap between donation and transplantation ethics, and routine human research ethics, by highlighting key ethical considerations and establishing normative standards for research involving donors.

The Guidelines comprise principles that are designed to be adapted for application in local jurisdictions, ensuring alignment with relevant national laws and existing ethical oversight. They are not intended to replace existing guidance, but rather to address gaps in guidance and reinforce the relevance of existing guidance. The principles highlight ethical considerations of particular relevance to research that intersects with routine practices relating to deceased donation for transplantation and should therefore be used in conjunction with routine ethical guidance for the conduct of human research in general and for deceased donation and transplantation of human organs, cells, and tissues. The guidelines do not provide practical advice on the implementation of principles. Further publications by the INTEGRITY Project team are planned that will provide explanatory content to assist in the interpretation and application of specific principles in particular research contexts.

While efforts were made to engage members of the public–as future possible donors- and donor family members and transplant recipients in the development of the Guidelines, these key stakeholders comprised a disproportionately small number of participants in the consultation and had limited involvement in the drafting process. Future revisions to the Guidelines should ideally be informed by a wider range of stakeholder perspectives from around the world.

### The core principles

In this section, we briefly discuss the significance of the eight core principles that provide the normative foundation for the INTEGRITY Guidelines and present their subsidiary principles in tabular format.

#### All research activities involving deceased donors should be consistent with respect for the dignity of human beings and their bodies

Principle 1 (see Table 1) draws attention to the specific ethical considerations of research involving deceased donors or deceased donation. Just as the WHO has designated human organs, cells, and tissues for transplantation “health products of an exceptional nature” [14], such research should be considered ethically exceptional. Many activities within scope of the guidelines may not directly involve a living person as a subject of ethical concern, for example when stored tissue specimens are used in biomedical research. In other research, a living person such as the recipient of a deceased donor transplant may be the focus of ethical concern, and consideration of the donor may be easily forgotten. The moral status and inherent ethical value of a living person are typically regarded as substantially greater than those of a deceased individual. Nevertheless, for many people, the treatment of the dead—including the body and its parts—retains important ethical significance.

**TABLE 1:** Principle 1.

Principle 1 1. All research activities involving deceased donors should be consistent with respect for the dignity of human beings and their bodies. 1.1 The involvement of deceased donors in research should be celebrated and acknowledged as a valuable gift and contribution to society comparable in significance to donation for transplantation. 1.2 Designated custodians of a deceased donor’s body, organs, cells or tissues should take responsibility for the respectful care of these materials throughout all research activities until they are transplanted into living persons, returned to donor families, stored, or appropriately disposed of. 1.2.1 Where feasible, the ultimate disposal of human remains should be in accordance with the known or expected wishes of the deceased and/or their family. 1.2.2 Where relevant, those making a decision about deceased donation of organs, cells or tissues in the context of research should receive information about the likely duration of storage of donated organs, cells or tissues, and about arrangements for the disposal of remains (see Principle 7). 1.3 Research activities should respect the cultural, religious, and spiritual needs of people and their families, facilitating where possible preferred practices relating to end-of-life care, the process of death, decision-making, and treatment of the human body before and after death. 1.4 When research activities involve interventions in the body of a deceased donor, care for the body of the deceased should be consistent with the respectful treatment of a deceased donor for transplantation. 1.5 If the body of a deceased donor is physiologically maintained or perfused by machines, e.g., mechanical ventilators or extracorporeal membrane oxygenation (ECMO) devices, for the purpose of research after death: 1.5.1 The family of the deceased should receive support consistent with that provided to the families of deceased donors for transplantation during the period leading to organ and tissue removal. 1.5.2 The family of the deceased should be permitted, where feasible, to attend the deceased. 1.5.3 The duration of time during which the deceased body is physiologically maintained or perfused should be strictly limited to the period necessary to achieve the relevant research aims, with careful consideration of the potential burdens of prolonged protocols on grieving families. 1.6 All research involving deceased donors should be subject to scientific and ethical review and oversight at an appropriate level. 1.6.1 Authorities with responsibility for overseeing human research, deceased donation, and transplantation activities in each jurisdiction should establish clear guidance and mechanisms to ensure that research involving deceased donors is reviewed appropriately by competent boards or committees, and that the principles outlined in these guidelines are upheld in the local context. 1.6.2 The level of oversight and review required may differ according to the nature of the research, the populations that may be impacted by the research, and/or the potential burdens or risk of harm associated with research activities. 1.6.3 Where unclaimed bodies or organs, cells, or tissues removed from unclaimed bodies after death may legally be used in research, there should be a specific process for ethical oversight of such use to avoid the exploitation of vulnerable individuals and communities. 1.7 When proposed research activities involving deceased donors are not addressed by current legal or ethical frameworks, such frameworks should, where possible, be revised to provide governance of these activities. 1.7.1 Where revision of existing frameworks has not yet occurred, exceptional arrangements for ethical review and oversight of proposed research activities may be implemented, provided that the proposed research adheres to these guidelines and complies with relevant laws and ethical standards governing deceased donation in the relevant jurisdiction. 1.8 Consistent with routine ethical safeguards for human research, when research involves deceased donors: 1.8.1 The scientific merit of proposed research should be evaluated to avoid unnecessary or futile research activities involving deceased donors. 1.8.2 A clear and independent mechanism(s) should be established to communicate with stakeholders, facilitate management of inquiries, and handle complaints or concerns about proposed or actual research activities.

This first principle accordingly establishes a requirement to consider the ethical dimensions of research involving donors regardless of whether the donor is imminently dying, recently deceased, or long dead, while allowing that particular ethical considerations may be more or less relevant depending on the time that has elapsed since death and the nature of research activities. The principle is important because most ethical guidance for research frames ethical considerations solely with a focus on how research may affect the living; in the United States of America, regulation of human research ethics notably excludes the dead [23]. Philosophers and others disagree on whether deceased individuals should be considered to have interests, and the extent to which living persons owe respect for such posthumous interests [25]. There is similar disagreement regarding the ethical obligations living persons may have to respect the known or estimated wishes of the dead regarding the treatment of their bodies, personal information, and material property. While laws sometimes provide a legal framework to govern the disposal of property, bodies, and even privacy of personal health data after a person’s death, there are often gaps. In many jurisdictions, for example, laws provide for the removal and use of tissues and organs after death in transplantation and research but lack guidance for those tasked with making decisions about involvement of a donor in research activities that may commence immediately prior to or following the determination of death [26].

Several of the subprinciples derived from Principle 1 reaffirm commonly accepted norms such as the importance of considering the cultural, religious and spiritual values and preferences of stakeholders in research, especially in the context of end-of-life care and decision-making. However, others address particular concerns that may be rarely encountered in other fields of research. For example, Principle 1.5.3 states that when the body of a deceased donor is physiologically maintained for the purpose of research, as was done in the Parsons’ model for xenotransplantation trials [5], the duration of such trials should be strictly limited. This aligns with the familiar principle of nonmaleficence in human research ethics which entails the minimisation of risks and burdens during research and limits the duration of trials or interventions. Extended utilisation of a deceased body in research requires careful assessment of the necessity of such use and proportionality of the benefits of research compared with the burdens on families, where relevant, and potential threats to the dignity of the deceased [22].

#### Research activities should be designed to provide equitable opportunities for individuals and communities to participate in, contribute to, and benefit from research involving deceased donors

The second principle (see Table 2) affirms the importance of justice in research, as demonstrated through equitable opportunities to participate in and benefit from research. Although this is a well-established principle of human research ethics, it merits inclusion as a core principle in these guidelines given the importance of equity in deceased donation and transplantation. Deceased donation for transplantation is often considered a public good, ideally conducted under the auspices of governmental health authorities who must uphold public trust by ensuring equitable access to the benefits of transplantation for the populations that contribute to donation.

**TABLE 2:** Principle 2.

Principle 2 2. Research activities should be designed to provide equitable opportunities for individuals and communities to participate in, contribute to, and benefit from research involving deceased donors. 2.1 Research should promote equity by ensuring fairness in the distribution of opportunities to be a research participant, of potential burdens and risks of research, and of potential benefits from research. 2.2 No individuals or groups should be systematically excluded from opportunities for involvement in research solely because of actual or perceived vulnerability. 2.2.1 To facilitate opportunities for inclusion of vulnerable populations in research, efforts should be made to address potential vulnerabilities, to reduce risks, and to remove barriers to participation in research activities. 2.3 When considering the benefits of research to which individuals and communities should have access, these should be defined as including the scientific knowledge gained or therapeutic products derived from research that may be used to prevent illness or disease progress, or to improve quality of care, e.g., for patients and their families, and/or to improve health outcomes 2.4 Where relevant, research involving deceased donors should strive to support equity in healthcare and health outcomes, consistent with the values that underpin deceased donation and transplantation programs. 2.5 Where possible, researchers and research institutions, donation and transplantation professionals, organizations, and registries, healthcare organizations, and other relevant organizations should cooperate and/or collaborate • to increase opportunities for research, • to improve efficiency in use of resources for research, • to maximise the relevance and value of research for diverse populations, and • to share information and knowledge for the benefit of all. 2.6 When data about deceased donation and transplantation activities are collected by donation or transplantation programs or by designated registries for non-research purposes and these data are not routinely made publicly available, mechanisms should be established to enable data sharing and to ensure equitable access to these data for research purposes.

Equitable inclusion in research is especially important if all members of the wider community are to benefit from scientific advances. Equitable representation in research activities supports a fair distribution of benefits and burdens and helps to produce therapies that are effective for all relevant populations [27]. Factors contributing to disparities in deceased donation for transplantation and access to transplantation and research participation, such as distrust among some communities, may be exacerbated when research intersects with donation activities [28].

Equity is also considered in the context of Principle 4, which focuses on the potential impact of research activities on the availability of and equity of access to transplantation rather than equitable inclusion in research and equity in the outcomes of research. Inevitably, there is some entanglement between the principles. In particular, Principle 4 addresses procedural justice in decision-making about research which has important implications for equity in access to transplantation as well as opportunities for research.

#### When designing, conducting, or evaluating a research study, ethical consideration should be given to the interests of all individuals who may be involved in or impacted by the research activities

Deceased donation is fundamentally relational, often involving complex connections between various stakeholders that require careful attention to ensure that relevant interests and the broader implications of actions or inactions are considered in decision-making. Exploring an opportunity for deceased donation, for example, can impact not only the possible donor but also their family, other patients requiring healthcare, transplant candidates, as well as public trust and future donation behaviours. When an opportunity for donation involves research activities, there may be additional or alternative stakeholders involved, and the key considerations in terms of potential benefits, burdens and risks for some or all stakeholders may change.

The scope of the third principle (see Table 3) may initially appear unfeasible, requiring an excess of ethical concern by researchers. However, it is important to note that consideration for the interests of all does not entail exhaustive exploration of every possible interest, nor does it require that all those potentially impacted must be directly involved in decision-making about research. Subprinciple 3.1 notably specifies that “primary consideration” should be given to those most involved in or impacted by research activities. The views of particular stakeholders may be more or less relevant when designing a particular study. Similarly, the level of detail required for consent for involvement in a study will differ according to the nature of research activities in which stakeholders are involved.

**TABLE 3:** Principle 3.

Principle 3 3. When designing, conducting, or evaluating a research study, ethical consideration should be given to the interests of all individuals who may be involved in or impacted by the research activities. 3.1 The interests of individuals or groups that are most involved in and/or impacted by research activities should receive primary consideration in the ethical design, conduct and evaluation of research. 3.2 The assessment of risks and potential burdens and benefits of research should take into account the perspectives and interests of all individuals and groups who may be affected. 3.2.1 When research interventions in deceased donors target specific organs, cells or tissues, the potential impact of these interventions on non-target (‘bystander’) organs, cells or tissues used, e.g., in transplantation, should be explicitly considered. 3.2.2 When research considerations influence the donation process and/or allocation of organs, cells or tissues donated for transplantation, the potential impact of these changes on the availability of and access to transplantation for individuals and groups should be explicitly considered. (See Principle 4.) 3.2.3 The likely impact of involvement in research on fulfillment of the deceased donor’s known or expected wishes regarding end-of-life care, donation for transplantation, contributing to research, or post-mortem treatment of their body should be considered when evaluating potential benefits, burdens and risks of research. 3.3 Key stakeholders should be included wherever possible in the design and ongoing oversight of research studies, e.g., donor families, transplant candidates or recipients, members of specific cultural communities, or donation and transplantation personnel, with appropriate management of potential conflicts of interests, where relevant. 3.4 Relevant donation agencies should routinely be consulted when designing studies that involve deceased donors. 3.5 Health professionals who are involved in deceased donation or transplantation activities but not engaged as researchers in studies that involve deceased donors should routinely be informed of research activities that may intersect with their clinical duties; they should have the opportunity to seek further information or assurance regarding the research and its potential impact on their practice. 3.6 Where health professionals are granted the right of conscientious objection with regards to performance of clinical duties that are inconsistent with their moral beliefs, this right should be extended to encompass clinical research activities with which they may be asked to assist. 3.6.1 When exercising the right of conscientious objection in the context of research involving deceased donors, health professionals should strive to ensure that their objection does not negatively impact opportunities for patients (or their families) to be involved in and/or benefit from research. 3.7 Research should only proceed when the expected benefits are judged to outweigh any unavoidable risks or burdens. 3.7.1 The risks and potential burdens for all those who may be negatively impacted by a research study should be avoided where possible and otherwise minimised as much as possible. 3.7.2 The standard supports and services provided to deceased donors, donor families, transplant recipients and healthcare professionals in the context of deceased donation for transplantation should also be provided when these individuals are involved in research, or when deceased donation occurs solely for the purpose of research. Additional or extended services may be necessary to ensure appropriate care is provided throughout the course of research activities.

#### Research activities should be designed in ways that safeguard the availability of donated organs, cells, and tissues and ensure equitable access to the benefits of donation and transplantation

Research activities have the potential to increase the availability of therapeutic products derived from donated organs, cells, or tissues [29–31]. For example, reconditioning or repair of donor organs prior to transplantation can improve rates of utilisation and/or graft outcomes [32]. However, inclusion of donated organs in some experimental studies could also risk non-utilisation or poorer outcomes if studies are unsuccessful. Research can also disrupt routine allocation systems if some transplant candidates or centres are not eligible, or unwilling, to participate in a study, potentially undermining equity in transplantation. Safeguarding the availability of donated organs, cells, and tissues for transplant and equity of access to the benefits of donation and transplantation is thus a core concern that must be carefully considered when evaluating the potential benefits and risks of proposed research. The principle does not preclude research that may temporarily disrupt routine allocation or even foreseeably decrease the supply of donated tissues or cells for transplantation, for example, where the longer-term benefits of research are expected to outweigh these harms.

Principle 4 (see Table 4) also highlights the importance of procedural fairness in institutional decision-making about research involving donors, which has implications for safeguarding donation for transplantation as well as equitable opportunities for research (as discussed in the context of Principle 2). Historically, there has been little transparency regarding the allocation of donated materials, typically those deemed unsuitable for use in transplantation, to researchers. Anecdotal reports suggest that in many countries, allocation decisions for research are often made *ad hoc*, in response to requests from research teams, which may favour those with pre-existing relationships with donation programs or personnel. More work is needed to help institutions and agencies establish procedures to govern these decisions, and those relating to direct involvement of possible donors in research prior to death, not least to improve transparency and accountability for key stakeholders such as donor and recipient communities. Further specification of principles to guide allocation decisions and policies when research activities may temporarily disrupt routine allocation systems, especially of organs for transplantation, may also be helpful.

**TABLE 4:** Principle 4.

Principle 4 4. Research activities should be designed in ways that safeguard the availability of donated organs, cells, and tissues and ensure equitable access to the benefits of donation and transplantation. 4.1 Efforts should be made to maintain the availability of organs, cells, and tissues for use in transplantation while supporting opportunities for involvement of deceased donors in research and use of donations in research. 4.2 Evaluation of the potential benefits and risks of proposed research involving deceased donation must consider the potential impact of research activities on the availability and equitable allocation of organs, cells, and tissues for use in transplantation. 4.2.1 Ensuring the availability of organs, cells, and tissues for use in therapeutic transplantation should usually take priority if there is a conflict between this goal and research goals that require the involvement of deceased donors in research or the use of donated organs, cells, or tissues. 4.3 Clear policies should be established by relevant institutions, in particular donation and transplantation agencies, to guide the management of requests for endorsement of or involvement in research studies, particularly those that require access to and use of deceased donor bodies, organs, cells or tissues, or data about deceased donors, donor families, or transplant recipients. 4.3.1 Policies should enable procedural fairness in decision-making about support for research projects and foster equity in access to donated organs, cells, and tissues for use in research and transplantation. 4.3.2 Representatives of donation authorities, deceased donor and transplant recipient communities, healthcare professionals, custodians of donor organs, cells or tissues, custodians of data in donation and transplant registries, and relevant research communities should be included in the development of policies and guidelines. 4.3.3 Prioritisation of research requests to make use of scarce resources (e.g., donated organs or tissues) or populations (e.g., deceased donors that may be involved in intervention studies) should be guided by the values and goals of relevant stakeholders, (e.g., donation programs and deceased donor communities). 4.4 Decisions about proposed research activities that may temporarily disrupt the routine allocation of organs, cells, and/or tissues for transplantation should be made transparently and clearly documented and disclosed to key stakeholders, including the public, which comprises the population of future deceased donors and transplant recipients. 4.4.1 Where relevant, research protocols should include a mechanism for monitoring and evaluation of the outcomes of any disruptions to routine allocation of organs, cells or tissues for transplantation. The results of this evaluation should inform future decision-making about research and about allocation of donated organs or tissues.

#### The privacy and confidentiality of personal information relating to deceased donors, donor families, transplant recipients, and, where relevant, healthcare professionals should be protected in all research activities

Principles 5 and 6 (see Tables 5 and 6) are consistent with routine ethical principles that stipulate protections for the privacy of research participants while advocating for transparency in research activities. In general, such considerations should also apply to research involving deceased donors, but they may sometimes be more complicated, especially when tensions arise between frameworks and policies governing data use and publication in research and non-research settings. Such tensions are discussed in the context of Principle 6 below.

**TABLE 5:** Principle 5.

Principle 5 5. The privacy and confidentiality of personal information of deceased donors, donor families, and transplant recipients should be protected in all research activities. 5.1 Appropriate mechanisms to ensure ethical governance of research activities that involve collection, storage, sharing or use of personal information should be implemented according to the nature of the activity(ies) and the context in which they occur. Such mechanisms should conform to privacy and data protection laws and standards in the relevant jurisdiction(s). 5.1.1 Specific provisions should be made by researchers and donation and transplantation registries for governance of data collected from Indigenous peoples to respect data sovereignty and the values and preferences of Indigenous peoples [33]. 5.2 Information about the collection, storage and use of data in donation and transplant registries should routinely be available to persons making decisions about deceased donation or transplantation; this information should address the possible use of data in research and, where relevant, options to access or limit use of data, withdraw data, or otherwise control data held in registries. 5.3 Access to and use of data held in donation and transplantation registries by researchers should be subject to ethical review and oversight, consistent with other forms of research involving deceased donors. 5.3.1 Registries must establish ethical guidelines for the evaluation of requests to access and use registry data in research. 5.3.2 Registries should maintain publicly available policies governing protection, use and access to data for research purposes. 5.3.3 The level of ethical review required for research involving use of data relating to deceased donation or transplantation activities will depend on several factors including: • the conditions under which data were collected such as consent provisions relating to use of data in research, • the nature of the proposed research including associated risks and potential benefits of data use, • the nature of the data that will be provided to researchers. 5.3.4 Some studies that use only aggregated and anonymised data in research may not require formal research ethics review, whereas others, e.g., those in which potentially sensitive population characteristics or outcomes are the focus of analysis, may require substantive evaluation. 5.3.5 When research involves use of data that are already in the public domain, such as registry data that may be reported in public or governmental websites, formal research ethics review may not be required; researchers and organizations that manage donation and transplantation data should nevertheless strive to ensure that relevant values and principles from these guidelines are applied in the design and implementation of their research. 5.4 Where relevant, research protocols should include plans for the management of personal information about deceased donors that may be discovered, and which could have significance for donor families, transplant recipients, or specific deceased donor communities. 5.4.1 Consent for research may include, where feasible and appropriate, tiered or dynamic consent mechanisms that would permit, for example, relevant individuals to be contacted in future to make a decision about disclosure of new findings or significant health-related information, or to make decisions about future uses of data or donated organs, cells, and tissues (see Principle 7) [34–36]. 5.5 Where specific research activities pose a significant risk of privacy breaches or loss of anonymity for individual donors, donor families, or transplant recipients, e.g., publication of case reports or highly innovative translational research, this risk should be clearly disclosed, and explicit consent should be obtained. (see Principle 7) 5.5.1 Additional safeguards should be incorporated in research protocols as necessary to manage risks to privacy, such as provision of guidance or professional support to transplant recipients or donor families involved in high profile studies relating to social media use, or management of interview requests from news media. 5.6 Care should be taken to ensure that necessary safeguards for privacy and data protection in research protocols do not create barriers to inclusion of populations that may benefit from research. 5.6.1 Deceased donation or transplantation programs that have low volumes of activity should not be prevented from conducting or reporting research involving small populations. Strategies should be used to address concerns about anonymity, e.g., with collaboration between programs or centres (see Principle 2) used to pool data and care in the reporting of results (see Principle 6).

**TABLE 6:** Principle 6.

Principle 6 6. All research activities must be conducted transparently and be open to scrutiny, to uphold public trust in deceased donation and in the integrity of research involving deceased donors. 6.1 Routine data collection is a fundamental safeguard of donation and transplantation programs and should be maintained throughout all research activities involving deceased donors. 6.1.1 Data relating to the involvement of deceased donors in research should be routinely collected and reported in national registries, consistent with the international standards that mandate the routine collection and public reporting of data relating to deceased donation for transplantation [14, 37]. 6.1.2 Where national registries do not yet exist, deceased donation and transplantation programs should routinely collect and regularly review information about research activities that involve deceased donors. 6.2 Appropriate mechanisms to ensure the transparency of research activities involving deceased donors should be implemented according to the nature of the research activity(ies) and the context in which they occur. Such mechanisms should conform to privacy and data protection laws and standards in the relevant jurisdiction(s). 6.2.1 A centralised, publicly accessible source of general information about research activities involving deceased donors should be established in association with equivalent sources of information about deceased donation for transplantation; this may provide links to other sources of more detailed information about specific research activities, e.g., online clinical trials registries or research project websites. 6.2.2 Researchers have primary responsibility for ensuring mechanisms are in place to maintain effective communication with research participants or other stakeholders in studies, and to effectively disseminate the findings of research to relevant stakeholder communities. 6.2.3 When the nature of research activities may delay or limit the disclosure of results or activities to stakeholders, e.g., due to the timeline of research protocols or commercial interests, these constraints on disclosure should be communicated to relevant stakeholders and carefully considered when evaluating the potential benefits and burdens of the proposed research. 6.3 Wherever possible, the results of research involving deceased donors should be: • published in scientific journals and communicated to relevant scientific and professional audiences to inform future research in which the involvement of deceased donors is considered. • made available to all relevant stakeholders, including donor families, using appropriate mechanisms to support their access to and understanding of research outcomes, and allowing for individuals to opt-out of such communications if desired. 6.4 When submitting results for publication, researchers should be required to attest that research involving deceased donors has been conducted in accordance with relevant ethical frameworks for research, and that any deceased donation activities involved in the research were consistent with the ethical standards outlined in the WHO Guiding Principles, [14] and the Declaration of Istanbul on Organ Trafficking and Transplant Tourism [38]. 6.4.1 The publication of research that investigates or documents unethical practices relating to deceased donation or transplantation, e.g., evaluating the outcomes of travel for transplantation that involves organ trafficking, is ethically permissible provided that the research itself adheres to relevant ethical standards. 6.5 Data about research activities should generally be reported in aggregate to reduce the risk of identification of individuals. 6.5.1 Where small numbers, e.g., in low volume donation or transplantation programs, increase the risk of individuals or groups being identified in data reports, consideration should be given to the omission of more granular results in publicly accessible reports. 6.6 When disseminating research findings to relevant audiences, efforts should be made to avoid the stigmatization of individuals and populations and to maintain respect for research participants even when they cannot be directly identified. 6.6.1 Efforts to avoid the stigmatization of populations - for example, when reporting on populations that have a higher probability of requiring organ transplantation and a lower probability of contributing to deceased donation of organs - should not discourage the transparent publication of research that reports specific challenges impacting specific groups, which is essential for informing efforts to address inequities. Concerns about the potential stigmatisation of populations should be carefully addressed at the stage of protocol refinement rather than considered only at the time of dissemination of results.

The use of health records data in research is increasingly an ethical concern, as the adoption of electronic records and artificial intelligence have expanded opportunities for research [39]. Data relating to deceased donors may present complexities in addition to those associated with use of routine health records data, or even use of data relating to deceased individuals, which is an emerging area of ethical interest [40]. For example, some data are collected for the purpose of donation for transplantation rather than therapeutic care of the donor, other clinical data may be collected after death, and some datasets will inevitably combine private information about individual donors as well as recipients, or donor family members as well as donors. These and other factors may introduce new ethical concerns or uncertainty, for example, regarding the purposes for which data may be used and by whom. Use of some data relating to deceased donors may therefore be considered beyond the scope of ethical guidance or legal frameworks established for use of ordinary health records data in research.

Principle 5 and its subsidiary principles affirm the importance of safeguarding privacy and protecting data relating to deceased donors and recipients of deceased donor transplants. The subprinciples stop short of providing nuanced guidance on specific questions of urgent practical importance in some settings, such as when consent may be waived for the use of registry data in research. Further work is needed to establish more specific guidance to support ethical access to and use of data in research activities.

#### All research activities must be conducted transparently and be open to scrutiny, to uphold public trust in deceased donation and in the integrity of research involving deceased donors

There is a particular concern for transparency in research activities involving donors and recipients of transplants from deceased donors, due to the need to maintain public trust in deceased donation and transplantation systems. Accountability for the safe and equitable procurement and use of donations, as well as avoidance of exploitation or other harms to donors during the end-of-life period, should be a concern not only for researchers but also for donation programs and personnel who may not be involved in research activities.

Deidentified data relating to deceased donation and transplantation are routinely reported in aggregate to the public at the national and international levels, although there is often less detail and transparency regarding utilisation of donated tissues and cells compared with organs used in transplantation [41]. However, the frameworks under which data are used and shared for the purpose of accountability or quality assurance in donation for transplantation may not routinely allow for the use of these data in research. Conversely, when publishing data collected or generated as part of research activities, specific protections may be required to minimise the risk of harm to individuals or communities. These considerations highlight a potential tension between the core principles: the protection of privacy may conflict with the requirement for transparency.

The principles make clear that the transparency and accountability considered standard for donation for transplant activities should be equally applicable when donation occurs for research or when donors are involved in research. In combination with Principle 5, Principle 6 and its subsidiaries provide a strong foundation at the procedural level by recommending that mechanisms to facilitate ethical governance and decision-making about data collection, storage, and use are needed.

#### Research activities should promote the autonomy of individuals who may be involved in or impacted by research

Principle 7 (see Table 7) stipulates respect for the autonomy of those involved in or impacted by research, and, in numerous subprinciples, articulates several important ethical considerations regarding recruitment for and decision-making about involvement in research. It thus addresses one of the foremost concerns of researchers and donation personnel: consent. Ethical and legal uncertainty regarding consent requirements in the context of particular types of studies involving donors has been widely discussed [21, 43–46]. Key questions include when consent for involvement in research should be obtained and from which stakeholders, and how much information should be provided and by whom.

**TABLE 7:** Principle 7.

Principle 7 7. Research activities should promote the autonomy of individuals who may be involved in or impacted by research. 7.1 Research activities should respect a person’s fundamental interest in • having control over their body and personal information, • having the option to be involved in decision-making that may impact them and, • where relevant, having substitute decision-makers make decisions in accordance with the person’s values and preferences. Recruitment 7.2 Individuals who are responsible for research recruitment or supporting decision-making about involvement in research should receive appropriate training. Core competencies of such individuals include being able to: • Understand relevant ethical considerations with respect to deceased donation and transplantation as well as research. • Provide appropriate care for the decision-makers and/or arrange appropriate referrals to other support services or care providers when necessary. • Support informed decision-making about research opportunities. 7.3 Potential conflicts of interest in the recruitment of donors, donor families, or transplant recipients for research, such as those that may arise as a result of holding dual roles as clinician-researchers, should be identified and carefully managed in accordance with relevant policies or guidelines. 7.4 Research protocols that involve recruitment of patients currently receiving clinical care -whether deceased donors or transplant recipients - should generally require coordination with relevant clinical teams or services. 7.4.1 Relevant donation and transplantation personnel should be familiar with current research activities and research coordinators should be familiar with relevant donation and transplantation programs, to avoid disclosure of misinformation and to ensure timely referral to donation, transplantation, or research coordinators, as relevant. 7.4.2 Where the recruitment of deceased donors for research is not under the auspices of donation personnel, this should routinely require coordination and consultation with relevant donation personnel or organizations. 7.5 In general, opportunities for deceased donation for transplantation should be introduced to relevant stakeholders prior to discussion of opportunities for involvement of deceased donors in research. 7.6 When decision-making about deceased donation for transplantation takes place, decision-makers should be informed of relevant opportunities for involvement of the donor in research. 7.6.1 If organs, cells, and/or tissues cannot be donated for use in transplantation, the option of donating these for use in research should be presented whenever possible. 7.6.2 When biological specimens and/or data relating to deceased donors for transplantation may be retained for the purpose of research, this should be anticipated and should usually be disclosed to individuals making a decision about donation for transplantation. 7.6.3 Where feasible and desired, it may be appropriate to offer people making a decision about deceased donation more choices about the distribution and use of donations for research purposes than are usually permitted when making decisions about deceased donation for transplantation. 7.7 Where options for involvement in research are present, for example if donated organs or tissues might be used in a range of research activities or transplant candidates might be eligible to participate in more than one clinical trial, individuals making a decision about involvement in research or donation for research should be empowered to choose from among the available options if they wish to do so. Authority for decision-making about involvement of deceased donors in research 7.8 Where necessary, legal frameworks should be revised and/or established to provide clear mechanisms for authorising the involvement of deceased donors in research. 7.8.1 In jurisdictions where advance care directives can be used to guide healthcare decision-making for people when they lose decision-making capacity, individuals should be enabled to express values or preferences about future involvement in research in such directives. 7.8.2 In jurisdictions that provide mechanisms for individuals to register their wishes or preferences with regards to donation for transplantation after death, e.g., by joining a donor registry, such mechanisms should include opportunities to express preferences or wishes regarding involvement in research or donation for research. 7.8.3 Where possible, the person(s) with legal authority for substitute decision-making about deceased donation for transplantation should also have authority to make decisions about a deceased donor’s involvement in research. 7.8.4 In some cases, legal authority for involvement of individuals in some types of research may appropriately reside with organizations or groups other than the individuals concerned or their substitute decision-makers [42]. 7.9 Public education about deceased donation should include both information about donation for transplantation and information about opportunities for individuals to be involved in research at the end of life or to donate organs, cells or tissues for use in research. Individuals should be encouraged to discuss their wishes regarding donation for transplantation and research with their families. 7.10 Clear and accessible guidance should be provided to substitute decision-makers regarding how decisions about involvement of donors in research should be made. 7.10.1 Guidance should address how to manage conflicts among members of donor families regarding the involvement of a deceased donor in research; this should be consistent with the approach to decision-making about donation for transplantation. 7.10.2 Subject to the laws in each jurisdiction, when making decisions about research activities that involve interventions in the body of a deceased donor before or after death, substitute decision-makers should prioritise the deceased donor’s known or expected values and preferences.
Consent requirements 7.11 To support voluntary and informed decision-making, research protocols (and protocols for decision-making about deceased donation that are inclusive of donation for research) should take into account: • The timing, frequency, and location of discussions about involvement in research, • The mechanisms used to provide information about the research, • The identity and role of the person involved in requesting or supporting decision-making about research and their roles and relationships with decision-makers, • The implications of accepting or declining participation in research on opportunities for transplantation or donation for transplantation, where relevant. 7.12 Where appropriate and feasible, the process of decision-making about involvement in research and/or donation for research should be designed to support dynamic and flexible decision-making, and to minimise confusion and burdens on decision-makers while ensuring that: • key information about the research and any practical implications that may be important are communicated and considered, • decision-makers are supported and given the option to explore or discuss further information throughout the process, • the limitations of information provided are made explicit (e.g., uncertainty regarding future uses of donated organs, cells, or tissues in research) • any options for or limitations on withdrawal from research are clearly communicated. 7.12.1 When designing or evaluating specific research protocols or broader systems for the recruitment of participants in research involving deceased donors, a range of consent models should be considered and adapted where necessary to provide the best fit in the local context, while satisfying legal requirements and the ethical obligations outlined herein. Disclosure of information 7.13 The mechanisms used to disclose information to decision-makers about research should be designed to optimise communication and understanding of information and to reduce the burdens of information disclosure. 7.14 The disclosure of information to decision-makers about involvement in research should be designed to ensure that key information is provided, considered, and tailored to meet the preferences and needs of individuals in the context of particular research protocols. Such information should typically include: • who is conducting the research, • the goals of the research, • what the research activities involve, • the potential benefits, burdens and risks of involvement in the research, and • how to obtain more information and/or express concerns or complaints about the conduct of the research. 7.14.1 Information about substantive risks or burdens of research involvement for individuals should routinely be explicitly disclosed and considered when seeking consent. 7.14.2 Information that is likely to be considered significant by donors, donor families or transplant recipients and/or could influence their decision-making about involvement in research should be routinely provided to those deciding about such involvement. 7.15 When involvement in research does not present substantive risks or burdens and decision-makers prefer not to receive detailed information about what research involves, it is not always necessary or appropriate to impose detailed information on decision-makers, provided that decision-makers have the opportunity to receive more information at the time of making the decision or in the future. 7.16 Some research may involve considerations that entail specific consent requirements including explicit disclosure of information. Information that is likely to be deemed significant by research participants and that should usually be disclosed during decision-making about research, where relevant, includes: a) The extent to which research activities may deviate from standard clinical practice in deceased donation or transplantation of deceased donor organs, cells, or tissues. b) The potential effects of research activities on deceased donation for transplantation or access to transplantation. c) The potential impact of research involvement on a donor’s end-of-life care or transplant recipient’s current or future clinical care, including the potential financial implications of any changes to care. d) Source(s) of research funding and potential commercial interests in the research, including the possibility of donated organs, cells or tissues becoming saleable products. e) The potential collection and/or use of genetic data. f) The potential creation of perpetual cell lines from donated materials. g) The potential use of donated materials in non-human animal research. h) The potential use of donated materials in research that may lead to the creation of children. i) The potential distribution of donated organs, cells, tissues, or personal data internationally, and/or transfer of custodianship of donated materials or data. 7.17 Potential assumptions on the part of research decision-makers that may be influenced by expectations of deceased donation for transplantation or of routine transplantation activities, and which may not be applicable in the case of some research activities should be explicitly addressed when obtaining consent. 7.18 Potential or actual conflicts of interest relating to research activities should be avoided, and unavoidable conflicts of interest should be disclosed. 7.19 When transplant candidates are offered organ, cell or tissue transplants that have been obtained from a deceased donor who was involved in an interventional study, they should receive any relevant information that may have clinical implications for their transplant. 7.19.1 If there are plans to collect or use data about the transplant recipient as part of a study that involves interventions in deceased donors, information about the study should usually be disclosed when offering the transplant, even if there is no information of potential clinical significance for the recipient. Waiver of consent and presumed consent
7.20 In some cases, an HREC Human Research Ethics Committee] or equivalent body may approve a waiver of consent requirements or deem it reasonable to presume consent for the involvement of individuals or groups in research activities. Factors that may influence whether it is ethically reasonable to presume consent include: • the relevant legal framework(s) in the jurisdiction, • the practical feasibility of seeking explicit consent, • the level of risk or potential burdens for those involved in the research, and whether these exceed the risks or potential burdens that they would otherwise encounter in their daily life or as part of their previously agreed involvement in donation or transplantation activities, • whether the intended involvement of the individual or use of their data or previously collected or donated organs, cells, or tissues in research is consistent with their known or expected values and preferences, • the presence of any information indicating that the individual or group would not wish to be involved in this type of research, or that they would wish to make an explicit decision about any such involvement.
7.20.1 It is generally reasonable to waive individual consent requirements for research involving use of data about deceased donors or transplant recipients that are routinely collected for the purpose of monitoring quality and safety, e.g., in registries, when the research aims to improve deceased donation and transplantation programs and their outcomes. Such secondary use of data should be consistent with the guidance outlined in Principle 6.
7.21 Although it may sometimes be reasonable to infer a person’s wishes regarding donation of organs, cells, or tissues for use in research on the basis of their previously expressed wishes regarding donation for transplantation, consent for - or refusal of - donation for research or involvement in research should not be presumed solely on the basis of expressed wishes regarding donation for transplantation.
7.21.1 In jurisdictions with an opt-out (‘deemed’ or ‘presumed’) model of consent for deceased donation for transplantation, consent or refusal for involvement of deceased donors in research should not be presumed solely on the basis of an individual’s decision to opt-out or not to opt-out of donation for transplantation, unless explicitly provided for in legislation.

Several authors have previously discussed the complexities that may arise when recipients of deceased donor organ transplants are involved in research due to the logistical challenges of providing relevant information and obtaining consent for research during decision-making about acceptance of a deceased donor organ offer [21, 44]. Researchers and donation personnel may be more anxious, however, about the implications of principle 7 and its subprinciples regarding decision-making about involvement of donors themselves in research. The families of prospective donors typically bear responsibility for decision-making about donation and donor involvement in research, where relevant. Donation decision-making often follows stressful and emotionally exhausting decision-making about end-of-life care and may itself be overwhelming for some families [47]; the additional cognitive load of information about research opportunities may be considered excessive. Some families may find detailed decision-making about donation or research distressing, and donation personnel and researchers may question the feasibility of informed decision-making in this context. Donation personnel may also worry that discussion of research opportunities could negatively impact consent for donation for transplantation.

The relevance of autonomy is also more complex when the individuals concerned are deceased, as occurs when decision-making about research takes place after the determination of death. Wicclair, for example, suggests that “it makes no sense to protect the autonomy of the dead” [48]. However, he also argues that respecting the known or estimated wishes and preferences of the dead with regard to involvement in research may be ethically justifiable. A key justification for doing so in the case of research involving donors is that this would be consistent with the approach to decision-making about deceased donation for transplantation.

Although legal frameworks in some jurisdictions allow consent to be presumed for the removal of organs for use in transplantation, in practice families (or equivalent) are routinely approached to make or confirm a decision about donation on behalf of the donor, assuming the donor is unable to do so. While family preferences sometimes override the known or estimated wishes of a possible donor, it is increasingly accepted that surrogate decisions about donation for transplantation should ideally follow a substituted judgment approach. That is, decisions should be based on what the possible donor would choose if they were able to make the decision [49]. Similarly, decisions about end-of-life care, such as the withdrawal of life-sustaining measures that may precede donation after circulatory determination of death, are increasingly made according to a person’s known or expected preferences and values, provided these are considered clinically appropriate and not disproportionately burdensome or harmful. It is thus reasonable to infer that decisions about donation for research, or involvement of donors in research during the perimortem period, should also be made using a substituted judgement approach where possible, as indicated in Principle 7.10.2.

The guidelines make clear the importance of tailoring consent processes and information disclosure to support effective decision-making, while minimising burdens and distress for decision-makers, whether they are possible donors providing first person consent, donor family members, or transplant candidates. Communicating key information for decision-making about research may require innovative strategies that differ from the detailed informed consent models commonly associated with clinical research or the blanket consent or “tick-box” options typically included in donation for transplantation forms to allow for use of donated materials in research.

The risks of relying on a “tick-box” option regarding involvement in research on the consent form for donation for transplantation should be self-evident: some decision-makers may decline important research opportunities due to lack of knowledge of the nature and potential value of the research, while others may agree without realising the extent to which research activities and their potential outcomes differ from those of routine donation for transplantation. In the absence of clear guidance regarding consent requirements and feasible mechanisms to support disclosure of information and decision-making about research opportunities, there is a risk that research may proceed without appropriate consent from or engagement with stakeholders, and/or that opportunities for valuable research will be missed. The principles outlined in these Guidelines provide grounds for the development of more specific guidance and practical advice regarding support for decision-making about research in particular settings.

#### Research activities must respect the prohibition of financial gain in the human body and its parts and uphold the principle of financial neutrality in donation

Finally, principle eight (see Table 8) reaffirms one of the core ethical tenets of donation and transplantation, which prohibits trade in the human body and holds that donors and donor families should neither lose nor gain financially as a consequence of donation [14, 38, 51, 52]. Regardless of the purpose for which donated organs, cells, or tissues are used–in transplantation or research–the underlying ethical concerns about financial interests influencing donation decisions, undermining equity, and exploiting the vulnerable remain relevant. The subprinciples outlined under principle eight do not preclude, where necessary and ethically appropriate, the provision of financial payment to living persons involved in research to cover the costs associated with their participation in research activities, or coverage of other research-related costs. Coverage of any costs associated with donation, whether for transplantation or research, is also consistent with the principle of financial neutrality [53, 54]. If a deceased donor’s involvement in research, for example, imposes additional financial burdens on their family, these should be alleviated wherever possible.

**TABLE 8:** Principle 8.

Principle 8 8. Research activities must respect the prohibition of financial gain in the human body and its parts and uphold the principle of financial neutrality in donation. 8.1 The prohibition of financial gain in the human body and its parts does not preclude the coverage or reimbursement of necessary costs associated with the conduct of research involving deceased donors, or the recovery, transport, preparation, preservation, storage or use of donated bodies, organs, cells, or tissues in research activities [50]. 8.1.1 Costs associated with research activities involving deceased donors should be routinely documented and open to scrutiny by relevant stakeholders. Financial neutrality in donation. 8.2 Deceased donors and their families should neither lose nor gain financially as a result of donation, whether donation occurs for the purpose of transplantation, research, or both. 8.3 Where additional costs are incurred by deceased donors, donor families, or transplant recipients because of their involvement in research, these should be covered whenever possible by relevant research, healthcare or governmental organizations. 8.4 Financial incentives, including fungible gifts or other rewards, should never be offered to individuals or families in exchange for their agreement to donate the body, organs, cells, or tissues after death for use in research, or to involve a deceased donor in a research study. 8.4.1 The principle of financial neutrality in donation does not preclude the use of incentives for recruitment of transplant recipients or donor family members in research studies, provided that: • routine ethical concerns regarding the use of incentives in research are appropriately addressed. • any incentives offered to donor families for their participation in research do not create actual or perceived conflicts of interest in donation decision-making, e.g., where their participation in research is contingent upon the donation decision.
8.5 Individuals with responsibility for recruitment of deceased donors for involvement in research should not be offered incentives or rewards proportionate to their success in obtaining agreement for deceased donation or involvement of donors in research. This does not preclude the payment of salaries or other costs associated with recruitment or research coordination activities.
8.6 Ceremonies and memorials that celebrate deceased donors who have contributed to research and/or transplantation are encouraged and should not be considered inappropriate incentives. Commercial interests
8.7 The presence of commercial interests in research involving deceased donors should be accepted where necessary for the success of research that offers substantial benefits for the public, especially through the development of new or improved medical products derived from human organs, cells or tissues, or the creation of valuable scientific knowledge relating to health.
8.7.1 When evaluating the potential benefits of research for the public, the potential impact of commercial interests on equity of access to these benefits should be carefully considered.
8.8 When proposed research activities involving deceased donors are intended or likely to generate financial gains for researchers, research institutions, commercial entities, funding agencies, or other parties, or when such gains become apparent in the course of research, relevant parties should be encouraged to invest a proportion of profits in a manner that: • Benefits public health, e.g., by supporting donation and transplantation programs, • Improves equity in access to healthcare resources, e.g., by subsidizing the costs of new medical products derived from donated organs, cells, or tissues for disadvantaged populations, and/or, • Supports research aimed at improving health outcomes for all.
8.9 The management of commercial interests in research activities requires scrupulous attention in the context of research involving deceased donors to avoid undermining public trust in the integrity, values and culture of deceased donation programs and personnel.
8.9.1 When potential, perceived, or actual commercial conflicts of interest relate to research activities that directly involve deceased donors, these should be • treated as significant risks when evaluating research, • routinely avoided where possible, • specifically disclosed to decision-makers about research, • routinely disclosed in publications reporting on research activities including reports by donation organizations or registries where relevant.

The prevailing ethos of donation for transplant is that of a public good, with equitable access to the benefits of deceased donor transplantation ideally ensured by public institutions. This may appear threatened when deceased donation intersects with commercial interests in research. In fact, commercial interests are already prevalent in the donation and transplantation sectors in many countries, especially in the context of the tissue sector [55]. While commercial interests may be necessary to bring innovative therapies to patients, such interests in research may raise concerns about the injustice of industry profiting from materials that are gifted without financial gain by donors. This is especially relevant if it is unclear or unlikely that the public will be able to freely or equitably access the benefits of the research, for example in the form of new and expensive medical products derived from donated materials. Addressing these concerns prospectively is important to maintain public support for donation and involvement in research; this requires particular care when research may involve the development of perpetual cell lines or use of genetic material [56]. The case of Henrietta Lacks, for example, has heavily influenced trust and attitudes towards use of biospecimens in research [57]. Further guidance will be required to address concerns in the context of specific types of research and jurisdictions. The subprinciples in this final section again provide high-level guidance that may be useful in grounding more specific principles or policy with regard to commercial interests in research involving deceased donors. In particular, the principles affirm the importance of upholding the fundamental ethical values embedded in donation for transplantation programs and highlight the need for caution and care to ensure that research activities do not jeopardise these values and the goals of such programs.

## Conclusion

In 2024, the World Health Assembly called on member states to “promote research and innovation to maximize the use and optimize the outcomes of transplantation of human cells, tissues and organs, as well as enable development of alternative therapies to those based on the clinical use of human cells, tissues and organs” [58]. These inaugural international guidelines provide a strong foundation for current research practice but require further work at the local level for adaptation and implementation, including training in their use for researchers, donation and transplantation professionals, and research ethics governance bodies. Further work is also required on specific issues at the normative and practical levels, including consent requirements and mechanisms, allocation issues, use of registry data, and the management of commercial interests in research.

Cultural change may also be needed within donation and transplant programs to address historical beliefs and practices regarding the role of research within the context of deceased donation activities. Where opportunities for involvement in research arise, these should be considered an option for donors and transplant recipients, rather than a competing interest. Timely consultation and collaboration between researchers and donation and transplantation programs can help integrate research opportunities into donation pathways efficiently, while maintaining high standards of care for donors, donor families and transplant recipients, and can help identify and address potential ethical concerns.

## Data Availability

The raw data supporting the conclusions of this article will be made available by the authors, without undue reservation.

## Group member of the INTEGRITY Project Collaborator Team

Juntaro Ashikari (Japan Organ Transplant Network, Division of Investigation and Research, Tokyo, Japan). Pacifico Calderon (St. Luke’s Medical Center College of Medicine-William H. Quasha Memorial, Manila, Philippines). Elena Cavazzoni (University of Sydney, Sydney, Australia), Celeste de Vaal (University of Cape Town, Cape Town, South Africa), Beatriz Domínguez-Gil (Organización Nacional de Trasplantes, Madrid, Spain), Rosalie Grivell (Australian Organ and Tissue Authority, Canberra, Australia), Daniel Harvey (NHS Blood and Transplant and University of Nottingham, United Kingdom), Marisa Herson (School of Medicine, Deakin University, Geelong, Australia), Alison Hodak (Australian Organ and Tissue Authority, Canberra, Australia), Georgina Irish (Adelaide University and ANZDATA Registry South Australia Health and Medical Research Institute, Adelaide, SA, Australia), Victoria Jennings (Deakin University, Geelong, Australia), Wenshi Jiang (China Organ Donation Administrative Center and Shanxi Provincial Organ Procurement & Allocation Center, China), Marta López-Fraga (European Directorate for the Quality of Medicines & HealthCare (EDQM), Council of Europe, Strasbourg, France), Heather Machin (Lions Eye Donation Service, Melbourne, Australia), Francisco Martinez (Division of The Skin & Tissue Bank, National Institute of Rehabilitation, Ministry of Health, and National Autonomous University of México, Mexico City, México), María Victoria (Martínez-López, Department of Nursing, University of Granada, Granada, Spain), Olivia Ngan (University of Hong Kong, Hong Kong, China), Jeffrey P. Orlowski (LifeShare Network, USA), Brendan Parent (Section of Medical Ethics, NYU Grossman School of Medicine, New York, USA), Rebecca Pentz (Winship Cancer Institute, Emory School of Medicine, USA), Alicia Perez Blanco (Organización Nacional de Trasplantes, Madrid, Spain Rizmina Rilwan, Independent Researcher Sibele Schuantes, Paulista School of Nursing, Federal University of São Paulo (UNIFESP) São Paulo, Brazil), David Thomson (Division of Critical Care, Department of Anaesthesia and Peri-operative Medicine, University of Cape Town, Groote Schuur Hospital, Cape Town, South Africa, Sandra Venter (Vitanova Tissue Bank, South Africa), Matthew J. Weiss (Transplant Quebec, Montreal, Canada), Matthew Wellberry-Smith (Leeds Teaching Hospitals NHS Trust and the University of Leeds, UK).
